# Effects of Dietary Bromide, Magnesium and Tryptophan and Immunocastration on Growth Performance and Behaviour of Entire Male Pigs

**DOI:** 10.3390/ani14243685

**Published:** 2024-12-20

**Authors:** Frank R. Dunshea, Ian McCauley, Robert J. Smits

**Affiliations:** 1 School of Agriculture, Food and Ecosystem Sciences, The University of Melbourne, Parkville, Melbourne, VIC 3010, Australia; 2 Faculty of Biological Sciences, The University of Leeds, Leeds LS2 9JT, UK; 3 Department of Primary Industries, 600 Sneydes Road, Werribee, VIC 3030, Australia; 4 Tri Advice Pty Ltd., 8 Clubbe Cr., Macgregor, ACT 2615, Australia; robsmits@triadvice.au; 5 Rivalea Australia, Corowa, NSW 2646, Australia

**Keywords:** magnesium, bromide, tryptophan, boar, immunocastration

## Abstract

The growth of boars may be inhibited because of aggressive and/or sexual activity. Dietary Br, Mg and tryptophan (Trp) as well as immunocastration may reduce these behaviours. In Experiment 1, 200 boars and 40 barrows were allocated to six groups of four pens of 10 pigs per treatment. Control and immunocastrate (Improvac-vaccinated at 13 and 17 weeks, Imp) boars and barrows were fed a finisher ration while the others were fed diets supplemented with Mg (5 g Mg proteinate/kg), Br (140 mg NaBr/kg) and Trp (5 g Trp/kg). In experiment 2, 300 boars were stratified by weight and within three weight classes allocated to two pens of ten pigs per treatment. Control and Imp boars were fed a finisher ration while the other diets were supplemented with Br, Trp or both Br and Trp. In Experiment 1, average daily gain (ADG) was not affected by diet but the Imp boars had higher ADG than controls. Feed intake (FI) tended to be higher in all treatments compared to controls except for the Trp group. In Experiment 2, Imp boars had higher ADG and FI than other treatments while Br+Trp boars had higher ADG and FI than controls. These data suggest that immunocastration and dietary Trp and Br show promise for improving performance in group-housed boars.

## Simple Summary

The growth performance of group-housed boars is well below that of their individually housed contemporaries and much of this difference in performance can be ameliorated by immunocastration. The improvements in performance after immunocastration are, at least in part, attributable to an increase in time spent feeding and a reduction in aggressive and sexual activities. However, a negative aspect is the increase in carcass fat. In-feed dietary additives such as bromide, magnesium and tryptophan offer another means to improve the performance of entire male pigs, although the effects do not seem to be as pronounced as immunocastration. Entire male pigs appear to be less motivated to feed than immunocastrates and less inclined to enter the feeder. Therefore, it may be important to ensure that feeder spaces are not limiting entire male pigs. Dietary sedatives may modify the behaviour of group-housed entire pigs and improve growth performance.

## 1. Introduction

Consumer preference is for pork from gilts or barrows rather than boars, and historically, male pigs have been castrated soon after birth in most parts of the world. However, in other regions such as Australasia, the United Kingdom and South Africa, male pigs have been kept entire, which has been purported to decrease the cost of production because of the better growth performance of boars compared to barrows. While this is certainly the case in male pigs experimentally housed in individual pens, the differences are not as pronounced when male pigs are housed in groups under commercial conditions [1,2,3]. For example, Suster et al. [2] found that over the final 4 weeks before slaughter, individually penned boars deposited 200 g/day more lean tissue than barrows. In contrast, there was no difference in group-penned animals. These differences are attributed to aggressive and sexual interactions between group-housed boars, which can be reduced by immunocastration [3,4].

Concerns about pig welfare issues surrounding castration have resulted in castration without anaesthesia being banned in some EU countries, with others likely to follow suit. For example, in 2002 the Norwegian parliament decided to ban castration from 1 January 2009, and until implementation of the ban, all castrations had to be performed under analgesia and by a veterinarian [5]. In 2010, 33 key stakeholders in the pork supply chain, including scientists, veterinarians and animal welfare organisations, voluntarily signed the European Declaration on Alternatives to Surgical Castration of Pigs. This agreement sought to end the practice of surgical castration of pigs without pain relief by 2012 and gradually phase out surgical castration entirely across the EU and European Free Trade Association (EFTA) countries by 2018 [6]. While this goal has not been completely achieved, some EU countries still desire to cease castration completely. In Australia, the Model Code of Practice for the Welfare of Pigs [7] recommends that alternative options that minimise or alleviate pain from elective husbandry procedures or the avoidance of their use should be adopted where possible. Therefore, the issues relating to sexual and aggressive activities of group-housed boars will continue to be a problem, particularly with regard to heavy-weight pigs.

Several researchers have attempted to modify the behaviour of group-housed pigs using dietary additives. For example, dietary tryptophan may raise brain serotonin and modify aggressive [8] or sleeping [9] behaviour in pigs. Furthermore, dietary magnesium supplementation has been demonstrated to reduce plasma catecholamine concentrations and the incidence of meat quality defects in negatively handled pigs [10]. Potassium bromide is a dietary neuroleptic that has been shown to decrease sexual and aggressive activities without altering the growth rate in growing bulls [11]. Also, dietary bromide has been found to increase [12] or have no effect [13] on growth in pigs. In the former study, there was an inhibition of sexual function or activity, which was reversed upon removal of the bromide from the diet. Therefore, it is possible that one or the other of these dietary treatments may be used to ameliorate the performance-detracting behaviours of group-housed boars. The aim of the present studies was to determine the growth performance of group-housed entire boars supplemented with dietary tryptophan, bromide and magnesium.

## 2. Materials and Methods

Both experiments were conducted at the Research and Development Unit (RDU) at Bunge Meat Industries (now Rivalea Australia Pty Ltd.) in Corowa, New South Wales, Australia. All procedures were approved by the Institutional Animal Ethics committee.

### 2.1. Experiment 1

#### 2.1.1. Growth Performance

The study involved 240 male pigs (in two replicates) comprising 200 entire boars and 40 contemporary castrate (surgical castration at 2 weeks of age) pigs. Pigs were allocated to treatment at 13 weeks of age and placed in 12 pens of 20 pigs (2 pens per subsequent treatment) in the RDU. The pigs destined to become immunocastrates were given the first dose of an immunocastration vaccine (Improvac, Zoetis Animal Health, Parkville, VIC, Australia) at 13 weeks of age. All pigs received a standard pelleted wheat and lupin based grower ration containing 14.0 MJ DE and 201 g crude protein per kg ad libitum until 17 weeks of age. Feed consumed and liveweight gain per pen of pigs were determined over the period from 13 to 17 weeks of age. At 17 weeks of age, each group of 20 pigs were divided into groups of 10 pigs (pre-determined at 13 weeks of age) and moved into pens in the finisher shed in the BMI RDU. Immunocastrated pigs were given their second dose of Improvac at 17 weeks of age and dietary treatments began. Control boars, immunocastrate and surgical castrate boars were offered a commercial pelleted wheat- and lupin-based finisher ration containing 13.3 MJ DE and 164 g crude protein per kg. The three other dietary treatments offered to entire boars were finisher diet supplemented with magnesium (5 g magnesium proteinate/kg, Mg; Lienert, Roseworthy South Australia 5371, Australia), bromide (140 mg sodium bromide/kg, Br; CSA Scientific, Port Adelaide, South Australia, 5015, Australia) and tryptophan (5 g tryptophan/kg, Trp; Kemin Industries, Killara NSW 2071, Australia). All diets were offered ad libitum and feed intake and liveweight were determined on a per-pen basis weekly. Pigs were slaughtered at 22 weeks of age and slaughter weight, P2 fat, leg fat and dressing percentage were recorded.

#### 2.1.2. Statistics

Growth performance over the late grower period between 13 and 17 weeks of age were analysed by ANOVA with sex group (Boar, Improvac or Castrate) as the main factors. All analyses were conducted using pen as the experimental unit. Growth performance over the finisher period between 17 and 22 weeks of age was analysed by ANOVA (Genstat for Windows 23rd Edition. VSN International, Hemel Hempstead, UK) with sex or diet group (control, Mg, Br or Trp boars, Improvac or castrate) as the main factors and replicate as a blocking factor. All analyses were conducted using pen as the experimental unit. Due to a mechanical failure at the abattoir, carcass weight was only obtained for the first replicate and so only these data have been used in the analyses of carcass weight and dressing out rate.

### 2.2. Experiment 2

#### 2.2.1. Growth Performance

The second experiment involved 300 male pigs (in two replicates) that were weighed and allocated to treatment at 13 weeks of age to evaluate the most promising treatments from Experiment 1, Br and Trp. Pigs were stratified by weight into three 33.3 percentiles and randomly allocated to one of five treatments. The pigs destined to become immunocastrates (60 pigs) were given the first dose of Improvac at 13 weeks of age. All pigs received a standard grower ration ad libitum until 17 weeks of age. At 17 weeks of age, pigs were placed in groups of 10 pigs of each weight × treatment group (pre-determined at 13 weeks of age) and moved into pens in the finisher shed. Immunocastrate pigs were given their second dose of Improvac at 17 weeks of age and dietary treatments began. Control boars and immunocastrate boars were offered a commercial finisher ration containing 13.3 MJ DE and 164 g crude protein per kg. The three other dietary treatments offered to entire boars were the finisher diet supplemented with bromide (140 mg bromide chloride/kg, Br), tryptophan (5 g tryptophan/kg, Trp) or both bromide and tryptophan at the dose levels. All diets were offered ad libitum, and feed intake and liveweight were determined on a per-pen basis weekly. Pigs were slaughtered at 22 weeks of age, and slaughter weight, P2 fat, leg fat and dressing percentage were recorded.

#### 2.2.2. Behavioural Measures

Direct measures of pig behaviour were taken on two occasions at 18 and 22 weeks of age. A total of 15 pens of pigs were observed by three trained people in each 50 min session. Each of the 3 observers spent 10 min of each 50 min session recording the behaviour of one group of 10 pigs at a time. At the end of 10 min, the observer moved to the next listed pen. Therefore, each observer recorded 5 pens of pigs over the 50 min session in the following order. Each day, every group of 5 pens was observed 3 times. Aggressive acts were defined as any incident involving two pigs where one or both pigs perform vigorous biting/slashing/pushing actions against the other, directed at any part of the body. Where a fight occurred between 2 pigs (i.e., there was reciprocal aggression), a bout criterion interval of 5 s was chosen to separate one bout from another. Where more than 2 pigs were involved, each pig was counted as having a separate bout. A mount was defined as the occurrence of a pig riding on the back of another pig, which may be standing, sitting or lying. As for aggressive activities, a 5 s bout length criteria was used to count a new act of mountings.

#### 2.2.3. Statistics

Growth performance over the finisher period between 17 and 22 weeks of age was analysed by ANOVA (Genstat for Windows 23rd Edition. VSN International, Hemel Hempstead, UK) with sex or diet group (control, Br, Trp, Br+Trp and Improvac-treated boars) and weight group (heavy, medium and light) as the main factors and replicate as a blocking factor. All analyses were conducted using pen as the experimental unit. Behavioural data were analysed by restricted maximum likelihood (REML) analysis, with the main effects being sex or diet group (control, Br, Trp, Br+Trp and Improvac-treated boars), weight group (heavy, medium and light) and week (18 or 22 weeks) as the main factors, and replicate, observer and sequence as blocking factors.

## 3. Results

### 3.1. Experiment 1

Castrate pigs were 1.7 kg heavier (*p* = 0.017) than contemporary boars at 13 weeks of age (Table 1). There was no effect of sex on daily gain between 13 and 17 weeks of age. Consequently, surgical castrates tended to maintain their weight advantage at 17 weeks of age (+2.3 kg, *p* = 0.098). Surgical castrates ate 20% (*p* = 0.005) more feed and used feed 16% (*p* < 0.001) less efficiently than entire male pigs over the period from 13 to 17 weeks of age. There was no effect of primary vaccination with Improvac on any aspect of growth performance until secondary vaccination. Over 17 to 22 weeks, the Improvac-treated boars grew more quickly than all other classes of pigs (Table 2). In particular, the immunocastrates grew 26% (+199 g/d) faster than the control boars. While the sedatives had no significant effects on daily gain, all group means were numerically greater (+4 to +9%) than that of the control boars, as was the case for the surgical castrates (+12%).

Feed intake of the castrates was higher than any other groups over the first 2 weeks of the finishing period (Table 2). There was no effect of any dietary additives on feed intake of entire boars over the latter part of the finishing phase. Immunocastrates increased their feed intake over the latter part of the finishing period to a similar level as the surgical castrates.

There was no effect of sex or dietary additives on feed conversion efficiency (FCR) over the first 2 weeks of the finishing period (Table 2). However, over the latter part of the finishing phase the FCR of the surgical castrate pigs was 21% higher than that of the control boars. Over the entire finishing period, the FCR of the Trp boars was 10% lower than that of the control boars, whereas the FCR of the surgical castrates was 17% higher than that of the control boars. The FCR of the Improvac-treated boars and the Br and Mg boars was not different from that of the control boars.

Carcass weight was significantly increased in the castrates, the Improvac-treated boars and the boars fed diets containing Br (Table 3). In the surgical castrates and Br boars, this resulted from increased live weight (Table 2) and dressing rate (Table 3), whereas for the Improvac-treated boars, the increased carcass weight resulted from increased live weight. Dietary Mg and Trp also increased the dressing out rate. Surgical castrates had higher P2 (+5 mm) and leg fat (+4.8 mm) than the control boars, whereas there was no significant effect of any dietary additives or Improvac on either P2 or leg fat.

### 3.2. Experiment 2

**Figure 1:**
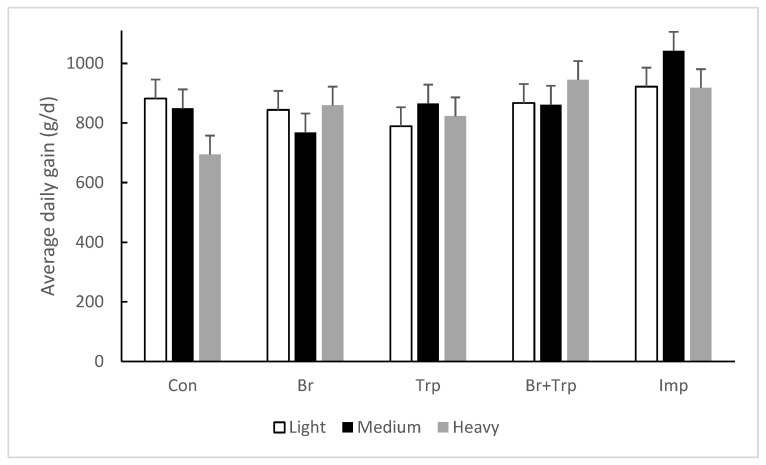
Effect of dietary neuroleptic or Improvac and weight class on average daily gain between 17 and 22 weeks of age. The error bars are the least significant difference (LSD) for weight × treatment = 134 g/d.

Growth data are presented in Table 4. There were no effects of treatment on liveweight at 17 weeks of age, demonstrating that prior injection with a single priming dose of Improvac had no effect on growth performance of boars. As planned, there were clear differences in the initial liveweight of pigs classed as heavy, medium and light (approximately 8 kg between each class of pig). Over the period from 17 to 22 weeks, the Improvac-treated boars grew more quickly than all other classes of pigs. In particular, the immunocastrates grew 19% (+153 g/d) faster than the control boars. However, there was an interaction between treatment and weight such that the growth response was greatest in the medium-weight class of pigs treated with Improvac and least in the light pigs (Figure 1). While there were no significant individual effects of either bromide (+1.8%) or tryptophan (+2.2%) treatments on daily gain, pigs treated with both compounds grew significantly faster (10.3%) than controls. Importantly, there was an interaction between treatment and weight class such that this effect was most pronounced in the heavy pigs where pigs from all treatment groups grew faster than the control boars (Figure 1).

Over the period from 17 to 22 weeks, the Improvac-treated boars ate more than all other classes of pigs, particularly over the latter three weeks of the study. Thus, over the entire 5 week treatment period, the Improvac-treated pigs ate 17% more feed than the control boars, whereas over the last 3 weeks of the study, the Improvac-treated boars ate 22% more feed than the control boars. While there were no significant individual effects of either bromide (+3.7%) or tryptophan (+2.9%) treatments on feed intake, pigs treated with both compounds tended to consume more feed (6.7%, *p* = 0.19) than controls. Indeed, over the first 4 weeks of the study, the pigs fed the combined Br+Trp diet consumed significantly more (+8.3%, *p* = 0.05) feed than the control boars. Light boars ate less feed than either the heavy or medium boars. There was no effect of any of the dietary or vaccine treatments on feed conversion ratio. There was a significant effect of pig size on FCR, with the lightest pigs using feed most efficiently and the heavy pigs being the least efficient.

Despite the differences in growth rate, there were no significant treatment effects on carcass weight or dressing percentage. There was no significant effect of dietary sedatives on any measures of backfat, although pigs fed the diet containing both Trp and Br tended to have a greater P2 backfat than the control boars (+1.0 mm, *p* = 0.06). However, this was principally due to the greater backfat depth in the heavy pigs compared to the other classes of pigs (+2.7, +1.2 and –1.0 mm for the heavy, medium and light pigs, respectively; LSD = 1.87 mm) as indicated by the significant interaction between treatment and weight. Improvac significantly (*p* < 0.05) increased ultrasonic backfat (+1.7 mm) and leg fat (+2.1 mm) and tended to increase slaughter P2 (+0.9 mm, *p* = 0.10). However, there was again an interaction, with the medium pigs treated with Improvac being fatter than the control boars, but not the heavy or light pigs (+1.0, +2.3 and –0.5 mm for the heavy, medium and light pigs, respectively; LSD = 1.87 mm).

Behavioural observations are presented in Table 5. The amount of time spent fighting, mounting or engaged in aggressive acts was not different between the treatment groups or between the weight categories. However, there was an increase in aggressive activity and mounting activity between weeks 18 and 22 of age. Feeder occupancy was significantly higher in the Trp boars and Improvac-treated boars than in the control boars or the boars fed diets containing both Trp and Br. Also, feeder occupancy was greater in pens of medium-sized boars than in pens of control boars. There were no treatments or weight group effects on the number of pigs queued for feeder space, but the number decreased with age.

## 4. Discussion

The important new findings from these studies are that dietary neuroleptics may also ameliorate the reduction in growth performance of commercially housed boars. While there were no significant main effects of Mg, Br or Trp on daily gain, in Experiment 1, all group means were numerically greater (+4 to +9%) than that of the control boars. In addition, dietary Trp significantly decreased FCR by 10%. A smaller overall effect was seen with bromide, but this became more pronounced throughout treatment and FCR declined to 2.67 in the last two weeks of growth. The Br pigs also tended to be heavier than entire boars at slaughter, with a heavier carcass and higher dressing. While there were no significant individual effects of either Br (+1.8%) or Trp (+2.2%) treatments on daily gain in Experiment 2, pigs treated with both compounds grew significantly faster (10.3%) than controls. Importantly, there was an interaction between treatment and weight class such that this effect was most pronounced in the heavy pigs, where pigs from all sedative groups grew faster than the control boars. A similar response was seen for feed intake.

Eidrigevich et al. [12] reported on several experiments involving growing pigs and fattening cattle administered daily doses of a Br/salt mixture. Their findings showed that a daily intake of 5 mg/kg of body weight, consisting of sodium, potassium and ammonium Br, enhanced the growth rate of the pigs. They also observed a temporary inhibition of sexual function during Br administration, but once the treatment ceased, both male and female animals could breed successfully. On the other hand, Barber et al. [13] found no significant effect of a mix of Br salts (ammonium, potassium and sodium), either alone or in combination with copper sulphate, on the growth performance or carcass characteristics of finisher pigs. The only other literature on the effects of Br on livestock was the work of Genicot et al. [11], who found that, while potassium Br supplementation did not affect ADG of Belgian Blue cattle, there was an improvement in feed efficiency (+9%) over the latter stages of treatment. Also, there were some behavioural alterations such that rear engagements and side and direct attacks were reduced during Br supplementation. Therefore, it appears that, under some circumstances, there may be some positive effects of Br in reducing sexual and aggressive activities in livestock with resultant improvements in growth performance.

The effects of Trp on pigs’ behaviour and growth have been much more studied than Br, particularly short-term studies focussed on pork eating quality. Some of these studies have also included Mg [14,15,16,17]. In general, there has been little or no effect on meat quality, although muscle pH has been increased in some cases, particularly in stress-susceptible pigs. Also, Peters et al. [15] found that dietary Trp-supplemented pigs were better able to handle simulated transport stress than their control counterparts. There have also been some long-term studies, and in one such comprehensive series of studies, Li et al. [18] found that supplemental Trp decreased the duration and intensity—but not the frequency—of aggression in unfamiliar finisher pigs. The pigs’ responses to handling stressors, including electric shock, were unaffected by Trp treatment. High dietary Trp did not affect growth performance or objective meat quality measures [18]. Polletto et al. [8] found that supplemental Trp increased blood Trp and serotonin concentrations and reduced aggressive behavioural activity and time spent standing while increasing lying. Supplemental Trp also reduced the number of agonistic interactions and aggressiveness in 3-month-old gilts. Dietary supplementation of Trp tended to increase ADG in 3-month-old gilts but not in 6-month-old gilts. More recently, Henry et al. [9] found that supplemental Trp increased plasma Trp and serotonin concentrations but did not affect ADG, feed intake or behaviour in weaner pigs. Therefore, as with Br, it appears that under some circumstances, there may be some positive effects of Trp in reducing sexual and aggressive activities in livestock, although it only occasionally results in improvements in growth performance. However, it should be borne in mind that none of these studies have used entire males, where aggressive and sexual behaviours are most pronounced.

Dietary Mg treatment between 2 and 5 days before slaughter has been demonstrated to reduce plasma catecholamine concentrations and the incidence of meat quality defects in pigs [10,19]. Subsequently, several short-term studies have shown some improvements in meat quality, particularly in stress susceptible pigs [14,15,16]. A recent systematic review of the effect of more long-term dietary Mg supplementation in pigs indicated that in most, but not all, studies, there were beneficial effects of dietary magnesium [20]. In the present study, the effects of long-term supplemental Mg were not as pronounced as the effects of Br and Trp, although ADG was increased over that of the control boars during the first 2 weeks of administration. These data are consistent with a transient increase in plasma Mg before declining to basal rates after 10 days of Mg feeding [19], meaning that the effects of dietary Mg supplementation may be short-lived. While there appear to be some positive effects of pre-slaughter dietary Mg supplementation on transport and lairage meat quality, the efficacy of longer-term Mg supplementation in reducing negative aggressive and mounting behaviour in entire male pigs is less compelling.

This study confirmed that surgically castrated pigs consume more and grow less efficiently over both the grower and finisher phases and are fatter at slaughter than entire and immunised males [21]. In turn, while there was no difference in growth performance between control and immunised entire male pigs over the grower phase, there was an increase in feed intake and ADG after the secondary immunisation particularly beyond 2 weeks after secondary immunisation. These findings are consistent with the literature as summarised in the meta-analysis of Dunshea et al. [22]. Although there seems to be clear evidence of immunocastration improving performance in group-housed boars, there is still reluctance in some quarters to accept the practice [23,24,25]. However, education of consumers may overcome some of these issues [24]. In Australia, for example, at least a 60% of male pigs are immunocastrated [26].

There were very few significant effects noted during the behavioural observations, perhaps because of the variation in behaviours or because the presence of observers may have impacted behaviour. When assessed using video analysis, there was a profound reduction in sexual and aggressive activities with immunocastration [4], but this was not observed here. Therefore, it is perhaps not surprising that there were also very few dietary effects on behaviour in the present study. Despite this, feeder occupancy was significantly higher in the Trp boars and Improvac-treated boars than in the other groups, suggesting that these animals were more inclined to enter the feeder. This is related to a greater fed intake, at least in the case of the immunocastrated male pigs.

## 5. Conclusions

Thus, it appears that the benefits of the combined sedative treatment were particularly pronounced in heavy entire male pigs, which are most likely to suffer a reduction in growth performance due to overcrowding and/or aggressive and behavioural activities (although this was not apparent from the limited behavioural observations). Further studies are required to determine the dose response and duration of treatment of these neuroleptic compounds and whether they can further enhance the beneficial effects of Improvac, the effects of which do not become pronounced until approximately 1–2 weeks after the secondary vaccination. In particular, NaBr is a relatively inexpensive compound, whereas Trp is relatively expensive. It is important to determine the most efficacious and cost-effective combination of these two dietary additives to improve the growth performance of finisher boars. It will also be important to understand the pharmacokinetics of NaBr to ensure that there are no issues with residues.

## Figures and Tables

**Table 1:** Effect of sex on growth performance over the late grower phase between 13 and 17 weeks of age in Experiment 1 ^1^.

	Boar	Improvac	Castrate	LSD ^2^	*p*-Value
Liveweight (kg)					
13 week	44.5	43.9	46.1	1.10	0.017
17 week	66.4	65.8	68.7	2.31	0.098
Growth performance					
Daily gain (g/d)	782	781	807	70.2	0.71
Feed intake (g/d)	1867	1823	2233	190.7	0.005
FCR (g/g)	2.39	2.34	2.77	0.139	<0.001

^1^ Improvac injections were given at 13 and 17 weeks of age. ^2^ Least significant difference (*p* = 0.05) between boars or Improvac-treated boars and castrate pigs. For least significant difference between boars and Improvac-treated boars, multiply by 1.265.

**Table 2:** Effect of sex and dietary additives on growth performance over the finisher phase between 17 and 22 weeks of age ^1^.

	Boars							
	Control	Mg	Br	Trp	Improvac	Castrate	LSD ^2^	*p*-Value
Liveweight (kg)								
17 week	66.1	66.5	67.8	64.5	65.1	68.5	2.91	0.093
19 week	76.0	78.4	78.8	75.4	77.9	81.1	3.08	0.037
22 week	93.7	95.2	97.9	94.1	99.8	99.3	4.66	0.044
Daily gain (g/d)								
17–19 weeks	709	851	790	776	915	900	175.8	0.162
19–22 weeks	826	782	889	879	1025	852	211.1	0.284
17–22 weeks	778	806	849	834	977	869	105.8	0.017
Feed intake (g/d)								
17–19 weeks	1876	2188	2209	1862	2187	2583	199.9	<0.001
19–22 weeks	2410	2381	2354	2314	3106	3076	323.8	<0.001
17–22 weeks	2201	2351	2335	2137	2738	2880	320.1	<0.001
FCR (g/g)								
17–19 weeks	2.67	2.66	2.85	2.43	2.41	2.87	0.518	0.308
19–22 weeks	2.99	3.07	2.67	2.63	3.15	3.62	0.487	0.006
17–22 weeks	2.84	2.95	2.77	2.56	2.95	3.32	0.196	<0.001

^1^ Improvac injections were given at 13 and 17 weeks of age. ^2^ Least significant difference (*p* = 0.05) between treatment groups.

**Table 3:** Effect of sex and dietary additives over the finisher phase between 17 and 22 weeks of age on carcass characterisitcs at slaughter ^1^.

	Boars							
	Control	Mg	Br	Trp	Improvac	Castrate	LSD ^2^	*p*-Value
Carcass weight (kg)	69.0	71.3	74.1	71.0	73.7	76.8	4.62	0.053
Dressing (g/kg)	751	761	761	760	755	773	8.70	0.009
P2 back fat (mm)	10.6	11.0	11.1	10.3	11.7	15.6	1.36	<0.001
Leg fat (mm)	13.7	12.6	13.5	12.9	15.1	18.5	2.32	<0.001

^1^ Improvac injections were given at 13 and 17 weeks of age. ^2^ Least significant difference (*p* = 0.05) between treatment groups.

**Table 4:** Effect of sex, dietary additives and liveweight on growth performance over the finisher phase between 17 and 22 weeks of age and carcass characteristics at slaughter ^1^.

	Treatment							Weight Class				
	Control	Br	Trp	Br+Trp	Improvac	LSD ^2^	*p*-Value	Heavy	Medium	Light	LSD ^3^	*p*-Value
Liveweight (kg)												
17 weeks	64.0	64.3	64.3	63.7	63.8	3.46	0.99	71.9	64.2	56.0	2.68	<0.001
18 weeks	69.0	69.7	69.8	69.8	68.7	4.04	0.96	77.3	68.9	61.9	3.13	<0.001
19 weeks	74.8	75.7	74.8	75.9	76.1	3.94	0.93	83.6	75.0	67.7	3.05	<0.001
20 weeks	80.5	82.0	81.0	82.4	84.1	3.96	0.37	89.9	82.3	73.8	3.07	<0.001
21 weeks	85.9	87.8	87.2	88.6	90.4	3.83	0.16	95.4	88.5	79.5	2.97	<0.001
22 weeks	92.3	93.1	93.2	94.9	97.5	3.98	0.093	101.6	94.9	86.1	3.08	<0.001
Rate of gain (g/d)												
17–19 weeks	771	812	751	871	873	189.9	0.55	832	777	838	147.1	0.63
17–21 weeks ^D^	781	803	820	889	949	70.7	<0.001	839	868	838	54.8	0.45
17–22 weeks ^D^	808	823	826	891	961	77.5	0.004	848	877	861	60.0	0.60
19–22 weeks	834	831	875	905	1019	107.4	0.012	859	944	876	83.2	0.10
Feed intake (g/d)												
17–19 weeks	2103	2162	2151	2205	2257	235.4	0.70	2354	2117	2056	182.4	0.008
17–21 weeks	2285	2354	2363	2475	2650	190.9	0.009	2550	2458	2268	147.9	0.003
17–22 weeks	2354	2441	2423	2511	2747	240.4	0.030	2579	2537	2370	186.3	0.069
19–22 weeks	2521	2627	2604	2714	3074	360.4	0.041	2728	2817	2579	279.2	0.22
Feed conversion ratio (g/g)												
17–19 weeks	2.89	2.71	2.91	2.54	2.59	0.562	0.54	2.87	2.81	2.51	0.435	0.20
17–21 weeks	2.96	2.93	2.88	2.80	2.80	0.259	0.59	3.06	2.85	2.71	0.200	0.007
17–22 weeks	2.95	2.96	2.94	2.81	2.86	0.227	0.58	3.06	2.90	2.76	0.176	0.009
19–22 weeks	3.09	3.16	2.98	2.99	3.02	0.436	0.89	3.21	3.00	2.95	0.338	0.24
Carcass characteristics												
Carcass weight (kg)	71.5	71.5	72.0	72.7	74.6	3.40	0.31	78.3	72.8	66.2	2.63	<0.001
Dressing (g/kg)	769.2	765.7	768.2	765.2	764.9	4.24	0.79	770.2	766.9	762.8	3.28	0.12
Ultrasound P2 (mm)	8.3	8.8	8.6	9.2	10.0	1.04	0.024	9.5	8.4	9.0	0.80	0.039
Slaughter P2 (mm) ^D^	9.3	10.2	9.5	10.3	10.2	1.08	0.18	10.4	10.2	9.2	0.83	0.016
Slaughter leg fat (mm)	11.6	12.0	11.3	12.3	13.7	1.14	0.004	12.3	12.8	11.4	0.88	0.015

^1^ Improvac injections were given at 13 and 17 weeks of age. ^2^ Least significant difference (*p* = 0.05) between treatment groups. ^3^ Least significant difference (*p* = 0.05) between weight groups. ^D^ Treatment × weight interaction (*p* ≤ 0.05).

**Table 5:** Effect of sex, dietary additives, liveweight and age on some direct behavioural observations in Experiment 2 ^1^.

		Treatment (T)					Weight (W)						Significance ^5^			
	Week	Control	Br	Trp	Br+Trp	Improvac	Heavy	Medium	Light	LSD ^2^	LSD ^3^	LSD ^4^	Treat	Weight	Week	T × W
Aggressive acts	18	9.5	10.7	14.2	9.2	11.7	11.0	8.8	13.4	4.98	3.85	3.15	0.23	0.35	0.025	0.18
(sec/10 min)	22	16.3	12.5	18.8	13.2	12.5	14.7	15.2	14.1							
Fights	18	6.3	4.6	8.4	12.5	8.6	6.5	10.5	7.3	4.31	3.34	2.73	0.54	0.16	0.39	0.90
(sec/10 min)	22	7.1	6.7	9.9	4.1	6.7	5.1	7.6	7.9							
Mounts	18	6.7	15.2	6.6	10.0	9.2	10.6	13.4	4.6	9.04	7.00	5.72	0.92	0.39	0.007	0.26
(sec/10 min)	22	17.1	12.4	18.6	21.8	16.8	21.4	13.2	17.5							
Feeder occupancy	18	0.76	0.74	0.86	0.65	0.82	0.72	0.78	0.79	0.103	0.080	0.065	<0.001	0.057	0.47	0.045
(pigs/feeder)	22	0.65	0.79	0.85	0.65	0.78	0.69	0.82	0.72							
Queued for feeder	18	0.40	0.35	0.38	0.34	0.43	0.32	0.43	0.39	0.128	0.099	0.081	0.57	0.59	0.040	0.098
(pigs/feeder)	22	0.23	0.23	0.40	0.34	0.28	0.29	0.28	0.31							

^1^ Improvac injections were given at 13 and 17 weeks of age. ^2^ Least significant difference (*p* = 0.05) between treatment groups. ^3^ Least significant difference (*p* = 0.05) between weight groups. ^4^ Least significant difference (*p* = 0.05) between week groups. ^5^ There were no other significant (*p* > 0.05) interactions.

## Data Availability

Data are available on request.
